# Field evaluation of a rapid immunochromatographic dipstick test for the diagnosis of cholera in a high-risk population

**DOI:** 10.1186/1471-2334-6-17

**Published:** 2006-02-01

**Authors:** Xuan-Yi Wang, M Ansaruzzaman, Raul Vaz, Catarina Mondlane, Marcelino ES Lucas, Lorenz von Seidlein, Jacqueline L Deen, Sonia Ampuero, Mahesh Puri, Taesung Park, GB Nair, John D Clemens, Claire-Lise Chaignat, Minoarisoa Rajerison, Farida Nato, Jean-Michel Fournier

**Affiliations:** 1 The International Vaccine Institute, Seoul, Korea.; 2 ICDDR,B: Centre for Health and Population Research, Dhaka, Bangladesh.; 3 CHAEM, Beira, Mozambique.; 4 Ministry of Health, Maputo, Mozambique.; 5 Médecins Sans Frontières, Geneva, Switzerland.; 6 National Institute of Child Health and Human Development, Bethesda, Maryland, USA.; 7 World Health Organization, Geneva, Switzerland.; 8 Institut Pasteur, Antananarivo, Madagascar.; 9 Institute Pasteur, Paris, France.; 10 Department of Statistics, Seoul National University, Seoul

## Abstract

**Background:**

Early detection of cholera outbreaks is crucial for the implementation of the most appropriate control strategies.

**Methods:**

The performance of an immunochromatographic dipstick test (Institute Pasteur, Paris, France) specific for *Vibrio cholerae *O1 was evaluated in a prospective study in Beira, Mozambique, during the 2004 cholera season (January-May). Fecal specimens were collected from 391 patients with acute watery nonbloody diarrhea and tested by dipstick and conventional culture.

**Results:**

The overall sensitivity and specificity of the rapid test compared to culture were 95% (95% confidence interval [CI]: 91%–99%) and 89% (95% CI: 86%–93%), respectively. After stratification by type of sample (rectal swab/bulk stool) and severity of diarrhea, the sensitivity ranged between 85% and 98% and specificity between 77% and 97%.

**Conclusion:**

This one-step dipstick test performed well in the diagnosis of *V. cholerae *O1 in a setting with seasonal outbreaks where rapid tests are most urgently needed.

## Background

The cardinal clinical feature of cholera is a severe dehydrating diarrhea, which can lead to severe and rapidly progressing dehydration and shock. Despite advances in the understanding of its pathophysiology and transmission, cholera remains a major international health concern. In 2003, the World Health Organization (WHO) received reports from 45 countries of 11,575 cholera cases and 1,894 related deaths. The majority of cholera cases occurred in sub-Saharan Africa [1]. However, these numbers are considered gross underestimates since outbreaks are often not reported due to fear of travel and trade sanctions. Critical interventions for cholera control include improved access to efficient treatment facilities, education to promote good personal hygiene, and improvement of sanitation and safe water supply [2-4]. But successful interventions depend on early detection of cholera outbreaks. Therefore, an efficient cholera surveillance system should be a high priority in the control of cholera [1,5].

The conventional culture methods currently used for diagnosis of *Vibrio cholerae *remain the gold standard but require a functioning laboratory and are time-consuming. Microbiologic facilities are usually not available in cholera-endemic settings, which are frequently characterized by abject poverty, or under emergency conditions such as natural disasters, wars, refugee crises, and population displacements. Thus, an accurate rapid bedside test would be of enormous help for the early confirmation of a cholera outbreak to enable preventive and control measures.

The Institute Pasteur, Paris, France has developed a one-step immunochromatographic dipstick test for the rapid diagnosis of *V. cholerae *from stool samples or enriched rectal swabs. This diagnostic test has been evaluated in Bangladesh and Madagascar, where it showed promising levels of sensitivity and specificity [6,7]. However, both evaluations were conducted in sites with a good research infrastructure. These findings cannot necessarily be extrapolated to sites in sub-Saharan Africa where most reported cholera cases occur. We therefore evaluated the Pasteur rapid cholera test in an endemic setting typical of many urban areas of Sub-Saharan Africa and used conventional bacteriological culture as the reference standard.

## Methods

### Study site and subjects

The port city of Beira in the province of Sofala is the second largest city in Mozambique. It has a population of approximately 450,000, divided among 22 districts (*bairros*). Diarrhea is highly seasonal in Beira and coincides with the rains that start in January, peak around April or March, and end about June. In Beira, the *Centros de Tratamento de Cólera *(Cholera Treatment Centre [CTC]) manages virtually all patients with watery diarrhea who require treatment. After evaluation of clinical signs such as dehydration, patients with severe dehydration are admitted and treated with intravenous (IV) fluids; patients with no or lesser dehydration are treated with oral rehydration solution (ORS) and observed at the CTC for 4 hours or longer. Diarrhea was defined as three or more loose bowel movements during a 24-hour period. Cholera was defined as a diarrhea episode during which *V. cholerae *were isolated.

For this study stool samples (bulk stool/rectal swab) were collected from consenting diarrhea cases who presented at the CTC between 1 January 2004 and 31 May 200. Stool samples were obtained by rectal catheter from patients with acute watery nonbloody diarrhea. If patients refused to have a rectal catheter inserted or the catheter failed to produce stool, a rectal swab was obtained. Rectal swabs were placed in Cary-Blair transport medium; bulk stool was transported in disposable plastic containers. All specimens were transported to a clinical laboratory within 2 hours of acquisition.

### Bacteriological culture

Conventional bacteriological culture was applied as gold standard against which we evaluated the accuracy of the IP rapid test. Bulk stool or rectal swabs were plated directly onto thiosulfate citrate bile salt sucrose (TCBS) agar and taurocholate tellurite gelatin agar (TTGA) [8]. In addition, the rectal swab was also plated onto TCBS and TTGA after enrichment in alkaline peptone water (APW) for 6 hours (pH 8.6, 37°C). After overnight incubation, suspected colonies on the agar plates were selected for biochemical test and agglutination with polyvalent, Ogawa, and Inaba antisera (Difco Formulation, Detroit, Michigan). Nonagglutinating strains were tested with antiserum to *V. cholerae *O139 strain (Difco Formulation, Detroit, Michigan).

### Dipstick test

The dipstick test utilizes monoclonal antibodies specific to *V. cholerae *O1 lipopolysaccharide (LPS) and colloidal gold particles based on a one-step, vertical flow immunochromatography principle. The detection threshold with purified LPS is 10 ng/ml for *V. cholerae *O1 [6].

In total, 200 μl of bulk stool was pipetted into a fresh tube into which the test strip was inserted. The rectal swabs were incubated for 6 hours in APW at 37°C after which 200 μl of the enrichment medium was also used for testing. The test strips were read after 10 minutes of immersion in the stool or in the APW suspension. The tests were defined as positive when both a test line and control line appeared on the test strip [6,7].

Technicians were trained for 2 weeks before the study start to perform conventional culture and the IP rapid test by a consultant from the Centre for Health and Population Research, Dhaka, Bangladesh (ICDDR,B) with prior experience in the use of the assay. The same technicians conducted the tests throughout the study period. The identity of 58 (42%) of 137 *V. cholerae *isolates were confirmed at the ICDDR,B.

### Data management and analysis

All information was double entered into a custom-made data entry program (FoxPro, Microsoft, Redmond, Washington, USA). The data management program includes both error and consistency checks.

The performance characteristics of the rapid test in different conditions, sensitivity, specificity, positive predictive value (PPV), Kappa coefficient, as well as 95% confidence interval (CI) for sensitivity and specificity were calculated [9]. Because of the close correlation between disease severity, intravenous rehydration and the type of specimen collected, the performance characteristics were assessed in four subgroups based on the specimen collected (bulk stool or enriched rectal swab) and treatment received (intravenous fluids or no intravenous fluids).

In the primary analysis, an intense test line (even in the absence of a control line) was considered as positive because it had been previously found that a heavy load of *V. cholerae *O1 LPS in the sample would cause most of the gold beads to bind with the LPS, resulting in an absent control line. In a secondary analysis we considered tests with an absent control line as invalid and excluded them from the analysis. We used the Chi-square test for analysis of binary data and a t-test for the continuous variables. To explore the potential correlation between the performance characteristics of the test and the type of stool specimen, the cholera prevalence and the experience of the technicians gained over time, firstly, we calculated the Pearson correlation coefficient for the type of stool specimen (rectal swab vs. bulk stool) and cholera prevalence (the percentage of culture-positive specimens of total specimens obtained biweekly). Subsequently, linear regression model was applied by treating sensitivity and specificity as response variables, and prevalence and proportion as independent variables. A p-value less than .05 (two-tailed) was considered statistically significant. For statistical analysis, we used an SAS program (version 8.2, SAS Institute Inc., Cary, NC, USA).

## Results

From 1 January to 31 May 2004, stool specimens from 391 patients were tested by conventional culture and by the dipstick test. Conventional culture detected *V. cholerae *more frequently in younger patients, in those with more severe clinical signs and symptoms who required IV rehydration, and in those who provided a bulk stool specimen (Table 1).

**Table 1:** Demographic and clinical features of diarrhea patients

Feature	Cholera cases (N = 138)	Non-Cholera diarrhea cases (N = 253)	p value
Mean (SD) age	20.2 (15.9)	24.4 (15.6)	<0.0001
Number (%) female	65 (47)	130 (51)	= 0.42
Number (%) rehydrated intravenously	104 (75)	84 (33)	<0.0001
Number (%) with severe dehydration ^a^	24 (18)	14 (6)	= 0.0002
Number (%) with vomiting ^b^	122 (88)	187 (75)	= 0.0012
Number (%) provided a rectal swab	66 (48)	153(61)	= 0.016

^a ^Data not available for 13 noncholeraic patients and 3 cholera patients.

^b ^Data not available for 2 non-cholera patients.

Overall, the sensitivity, specificity, PPV, and Kappa value were 95% (95% CI: 91%–99%), 89% (95% CI: 86%–93%), 83%, and 0.82 (p < 0.05), respectively. We compared the performance of the rapid dipstick by type of fecal specimen tested (219 rectal swabs, 172 bulk stool specimens). The overall sensitivity and specificity of the rapid test with rectal swabs were 97% (95% CI: 93%–100%) and 97% (95% CI: 95%–100%), respectively, and the PPV was 94%. In contrast, the sensitivity, specificity, and PPV of bulk stool were 93% (95% CI: 87%–99%), 77% (95% CI: 69%–85%), and 74%, respectively. The rapid dipstick had a higher sensitivity when rectal swabs were examined after 6 hours enrichment compared to bulk stool specimens irrespective whether the patients were or were not treated with IV fluids (table 2).

**Table 2:** Stratified analysis of the sensitivity and specificity of cholera dipstick test according to specimen (bulk stool or enriched rectal swab) and treatment (intravenous fluids or no intravenous fluids)

Dipstick test	Bacteriological culture		Total
		
	Positive	Negative	
IV treatment – enriched rectal swab ^a^			
Positive	45	3	38
Negative	0	40	40
Total	45	43	78

IV treatment – bulk stool ^b^			
Positive	57	10	67
Negative	2	31	33
Total	59	41	100

No IV treatment – enriched rectal swab ^c^			
Positive	19	1	20
Negative	2	109	111
Total	21	110	131

No IV treatment – bulk stool ^d^			
Positive	10	13	23
Negative	3	46	49
Total	13	59	72

NOTE: IV = intravenous fluids were given.

^a ^Sensitivity was 100%; specificity was 93% (85–100); positive predictive value was 94%; and Kappa value was 0.93 (p < 0.05).

^b ^Sensitivity was 97% (92–100%); specificity was 76% (62–89%); positive predictive value was 85%;and Kappa value was 0.74 (p < 0.05).

^c ^Sensitivity was 91% (78–100%); specificity was 99% (97–100%); positive predictive value was 95%;and Kappa value was 0.91 (p < 0.05).

^d ^Sensitivity was 77% (54–100%); specificity was 78% (67–89%); positive predictive value was 43%;and Kappa value was 0.42 (p < 0.05).

Of the 391 patients from whom specimens were tested, 188 received IV fluids. If only patients who required IV rehydration were considered, the overall sensitivity of the rapid test was 98% (95% CI: 95%–100%), specificity was 85% (95%CI: 77%–92%), and the PPV was 89%. In contrast, the overall sensitivity and specificity among patients who did not require IV rehydration were 85% (95% CI: 73%–97%) and 92% (95% CI: 88%–96%) and the PPV was 67%. The rapid dipstick had a higher sensitivity when specimens from patients receiving IV therapy were examined than specimens from patients who did not receive IV therapy irrespective whether bulk stool or enriched rectal swabs were examined (table 2).

**Figure 1:**
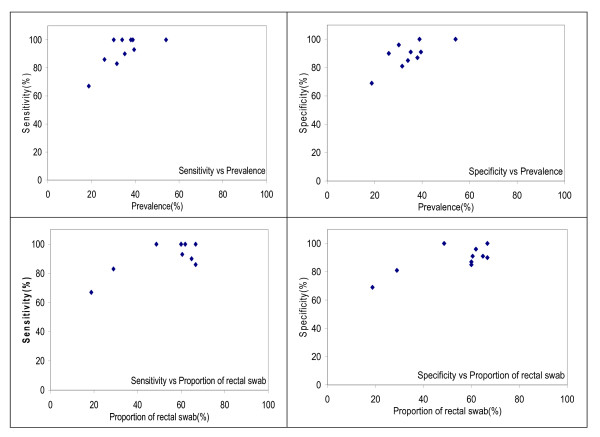
Sensitivity and specificity of the rapid dipstick test by month as related to cholera prevalence (% of tested specimens positive) and % of specimens that were rectal swabs.

The performance characteristics of the rapid tests improved over time (Figure 1). During the first month of testing, the sensitivity and specificity were 80% and 77%, respectively. The sensitivity and specificity for the subsequent 4 months increased to 97% and 92%. The performance of the rapid tests peaked in March when sensitivity and specificity were 100% and 92%, respectively. Correlation was observed not only among the sensitivity, specificity, proportion of rectal swab and cholera prevalence but also between the specificity and time (p < 0.05). However, significant correlation was only found between the cholera prevalence and the specificity (p = 0.04) when linear regression model was applied. The residual diagnostics did not provide any strong evidence for the violation of linearity and normality assumption for the linear regression model.

Of 391 tests, 52 tests lacked control lines but had strong test lines. If these tests were excluded from the analysis, the overall sensitivity, specificity, PPV and Kappa value based on the remaining 339 tests were 92% (95% CI: 86%–98%), 90% (95% CI: 86%–94%), 76% and 0.76 (p < 0.05) respectively.

## Discussion

This simple, one-step dipstick test performed well in the diagnosis of cholera in a setting with minimal facilities, where rapid tests are most urgently needed. Diagnostic testing may not be necessary for the clinical management of each diarrhea patient during an outbreak. The dipstick test may be helpful to confirm clinically suspected cholera cases, especially during the start of an outbreak. Once a cholera outbreak has been confirmed, large-scale preventive measures, including mass vaccinations and improvement of water and sanitation could be mobilized to minimize morbidity and mortality [3,5].

In our study, the overall sensitivity and specificity of the dipstick test were 95% and 89%, respectively. The performance of IP rapid test varied with the severity of disease and by type of specimen tested. The test was more sensitive for specimens from patients with life-threatening cholera who required IV rehydration than for specimens from patients with less severe disease. Patients treated with IV fluids may have more severe disease and their stool specimens may have a higher bacterial concentration than specimens from patients with milder illness [10,11]. The test had higher sensitivity as well as specificity after enrichment of rectal swabs in APW for 6 hours compared with direct immersion in bulk stool. The most likely explanation is that the use of Cary Blair transportation medium and the 6 hours enrichment may increase bacterial concentration of rectal swab. Because of the close correlation between the type of specimen tested (bulk stool or enriched rectal swab) and the treatment received by the patient (IV or no IV rehydration) we compared the performance characteristics of the rapid test in four subgroups. The subgroup analysis indicated higher sensitivity of the rapid test in IV treated patients with presumably more severe disease than patients with less severe disease, not requiring IV rehydration. The explanation for these observations may lie in differences in bacterial load. The enrichment of stool specimens derived from rectal swabs may increase bacterial concentrations to higher levels than bacterial concentrations found in bulk stool specimens. Specimens from patients receiving IV therapy may contain a higher bacterial load than specimens from patients not receiving IV therapy. The rapid test is least sensitive for mild cholera episodes, which are most difficult to recognize clinically.

In this study, the test performed less well during the first month of the study, likely because of incorrect use of and interpretation of results. We included the early test results in the overall evaluation of the dipstick test because we believe it is important to understand the dynamic character of the performance characteristics of the rapid test. It can be expected that the initial use of the rapid test will produce less satisfactory results than during routine use of the test. Since the first use of the assay may be critical in the detection of a cholera outbreak, cautious interpretation of initial test results may be indicated.

The absence of a positive control line in the presence of a very strong positive test line is likely related to heavy load of *V. cholerae *O1 LPS. Ideally, tests with absent control lines and strong test lines should have been retested after dilution of the fecal specimen. We did suggest adding the dilution step to resolve results which are difficult to interpret. However, under real field circumstances this is unlikely to be done because of additional time and manpower requirements. An alternative approach, interpreting all tests with an absent control line as invalid, probably results in an unacceptable decline in the sensitivity and PPV of the test. In practice, study staff found it easy to recognize strong positive test lines even in the absence of control lines and to interpret them correctly. The problem has been discussed with the manufacturer and hopefully will be resolved in future generations of the test.

Several rapid diagnostic tests based on monoclonal antibodies against *V. cholerae *O1 or O139 have been evaluated [4,12-16]. Though the sensitivity and specificity of these tests exceeded 95%, these assays are more complicated than the dipstick test and may not be suitable for use in the field [14,16]. In the earlier studies, the sensitivity and specificity of the dipstick test were 94%–100% and 84%–100%, respectively, very similar to the characteristics we found after the first month of study. The marginally better performance of the test in earlier studies may be explained by the more sophisticated laboratory infrastructure as well as the different disease spectrum in earlier studies [6,7].

In our study, the results of dipstick assay could be interpreted objectively with stool culture as the reference standard. Thus, other common confounders or biases of diagnostic studies, namely influence of clinical factors on test interpretation and reference standard error were unlikely to affect the validity of our evaluation [17,18].

## Conclusion

In conclusion, the dipstick test for detection of *V. cholerae *O1 is an accurate and easy-to-perform assay that does not need special equipment. It remains unknown how well the test performs directly in the hands of primary healthcare providers at a patient's bedside. We expect the performance will be similar as our findings during the first month of our study. Further studies are indicated to evaluate this assay in crisis situations, but the logistics may make such an undertaking even more challenging.

## Competing interests

The author(s) declare that they have no competing interests.

## Authors' contributions

ML, JLD, CLC and JDC contributed to the study's conception and design. XYW, RV, CM, JLD, LVS and SA carried-out the study and supervised the study staff. MKP created and installed the data management system and supervised its use. MA trained the laboratory staff and assured the accurate identification of *Vibrio cholerae*. XYW, LVS and TSP analyzed the data. XYW, JLD, LVS and JDC wrote the paper. GBN, MR, FN, JMF and CLC provided important suggestions in the writing of the paper. All authors read and approved the final manuscript.

## Pre-publication history

The pre-publication history for this paper can be accessed here:


